# Diagnostic dilemma of celiac disease presenting with weight loss and secondary amenorrhea: A case report

**DOI:** 10.1097/MD.0000000000031350

**Published:** 2022-10-21

**Authors:** Arif Mumtaz, Qaisar Ali Khan, Nowshad Asim, Abdul Baqi, Sumaira Iram, Abdul Majeed, Muhammad Junaid Tahir, Md. Saiful Islam, Zohaib Yousaf

**Affiliations:** a Khyber Medical University, KMU-IMS, Kohat, Pakistan; b Mercy Saint Vincent Medical Center, Toledo, OH, USA; c Sultan Qaboos University and Hospital, Oman; d Liaquat College of Medicine and Dentistry, Karachi, Pakistan; e Lahore General Hospital, Lahore, Pakistan; f Department of Public Health and Informatics, Jahangirnagar University, Savar, Dhaka, Bangladesh; g Centre for Advanced Research Excellence in Public Health, Savar, Dhaka, Bangladesh; h Hamad Medical Corporation, Doha, Qatar.

**Keywords:** anemia, celiac disease, gastroenterology, intestine, iron deficiency, medicine

## Abstract

Celiac disease (CD) is autoimmune enteropathy affecting the proximal small intestinal mucosa. It is caused by insensitivity to gluten, a protein predominantly presented in wheat. CD is classically associated with gastrointestinal symptoms. The non-classic clinical presentation of CD can present with other organ involvement. Non-human leukocyte antigens genes are associated with atypical forms.

**Patients concern:**

We reported a case of 30-year-old female who presented with progressive pallor, amenorrhea, and unexplained weight loss with generalized body weakness. Her body mass index was 20. The patient was having no other systemic manifestations.

**Diagnosis:**

This paper reports a case of a female patient having CD without its typical features. Her laboratory evaluation revealed microcytic anemia. Anti-TTg IgA and Anti-TTG IgG antibodies were raised, ferritin and folate were low, and there was mild hyperbilirubinemia. However, follicle-stimulating hormone, luteinizing hormone, and serum estradiol levels were normal. She was diagnosed with a case of anemia resulting from malabsorption caused by CD.

**Interventions:**

A management plan was devised based on a strict gluten-free diet. The patient received supplements containing folates, iron, calcium, zinc, and vitamins A, D, E, B6, and B12.

**Outcomes:**

After 3 months of treatment with strict gluten-free diet patient showed remarkable improvement. Her hemoglobin level raised with weight gain. Her normal menstrual cycle was restored with complete resolution of symptoms at 1 year follow-up.

**Lessons:**

The pathogenesis of the atypical CD is multifactorial, but impaired uptake of micronutrients from the duodenum is the most likely cause, even if other common features of classical forms, such as bloating and diarrhea, are absent. Lack of awareness about atypical forms may lead to under-diagnoses of the disease. The physicians should consider the atypical presentations of CD to avoid the under-diagnoses of this multisystem disorder.

## 1. Introduction

Celiac disease (CD) is a common disease and affects about 1 in 250 people in the United States and approximately 1% of people worldwide.^[1]^ CD is an inflammatory autoimmune disease of the small intestine due to genetic, immunological, and environmental factors. The atypical clinical manifestations of CD may affect up to half of all diagnosed patients. Prior to being diagnosed with CD, patients may develop hematologic issues. Few or no gastrointestinal symptoms may be present in atypical forms. Extra-intestinal manifestations are predominantly dermatologic, endocrine, hematologic, hepatic, psychologic, neurologic, reproductive, renal, and musculoskeletal.^[2]^ The malabsorption of iron, folic acid, and vitamin B_12_ can lead to anemia.^[3]^ Iron-deficiency anemia is common in newly diagnosed patients and can persist despite a gluten-free diet (GFD).^[4]^ The prevalence of anemia is < 1% in adult males < 50 years, 2% to 4% in males above 50 years, 9 to 20% in menstrual teenagers and young women, and 5% to 7% in postmenopausal women.^[5]^

The prevalence of CD in females, especially during the fertile period, is high.^[4]^ Delayed menarche, early menopause, amenorrhea, infertility, recurrent spontaneous abortions, and osteoporosis have been frequently seen in these patients.^[6,7]^ Underdiagnosis or untreated CD can lead to the development of long-term sequelae.^[8]^

## 2. Case report

A previously fit and well 30-year-old female patient from District Kohat Khyber-Pakhtunkhwa, Pakistan, was presented at the outpatient department (OPD) with progressive unintentional unexplained weight loss of 10 kg from the past few months associated with amenorrhea, fatigue, and exertional shortness of breath. She also complained of having myalgias and generalized weakness for 3 years. Her body mass index was 20. The patient was married and had 2 children with the last delivery 5 years ago. She had no history of miscarriages. There was no history of bleeding, joint pain, oral ulceration, hair fall, Raynaud phenomenon, rashes, epigastric pain, jaundice, and pruritis. A review of systems was otherwise unremarkable. Past medical history was insignificant except for occasional non-steroidal anti-inflammatory drugs used for body aches.

Upon examination, the patient was alert and oriented. Pallor was seen with leukonychia and koilonychia. There were no neck swelling or enlarged lymph nodes. The rest of the clinical examination was unremarkable.

Initial investigations indicated microcytic hypochromic anemia, and elevated erythrocyte sedimentation rate (Table 1). A peripheral smear showed microcytes with anisocytosis. Further workup revealed low ferritin, low folate and mild hyperbilirubinemia (Table 2).

**Table 1:** Complete blood count.

Test	Result	Units	Reference range
Hemoglobin	7.8	g/dL	14–18
Mean corpuscular volume	69	fL	80–98
Total leukocyte count	3800	mil/mcL	4000–11,000
Platelets	220,000	mil/mcL	150,000–450,000
Red blood cells	3.5	mil/mcL	4.2 - 5.4
ESR	55	mm/hr	<20
RDW	28.5	%	12.2-16.1
Mentzer index	19.7	–	-

ESR = erythrocyte sedimentation rate, fL = femtolitre, g/dL =grams per deciliter, hpf = high power field, mil/mcl = millions per microliter, mm/hr = millimeters per hour, RDW =red cell distribution width.

**Table 2:** Laboratory investigations.

Test	Result	Units	Reference range
Ferritin	3	ng/mL	11–306
Folic acid	1.5	ng/mL	4–20
Albumin	3	g/dL	3.4–5.4
Calcium	7.5	mg/dL	8.6–10.3
TSH	3.5	mlU/L	0.38–4.31
Free T4	1.1	ng/dL	0.7–1.8
Alanine transaminase	64	U/L	7–56
Alkaline Phosphatase	107	U/L	30–120
Total Bilirubin	2.1	mg/dL	0.1–0.3
Antinuclear antibodies	Negative		
Anti-TTg IgA	117	U/mL	<8.0
Anti-TTG IgG	169	U/mL	<10.0
FSH	4.2	mIU/mL	1.5–12.4
LH	3.4	IU/L	2–9
Serum Estradiol	40	pg/ml	30–400
Vitamin B12	340	pg/ml	160–950
Heptaglobin	195	mg/dL	50–200
LDH	165	U/L	140–280
Coomb test	Negative	–	–

Anti-TTG IgA = anti-tissue transglutaminase IgA, Anti-TTG IgG = anti-tissue transglutaminase IgG, FSH = Follicular stimulating hormone, g/dL = grams per deciliter, IU/L = international units per liter, LH = luteinizing hormone, LDH = lactate dehydrogenase, mg/dL = milligrams per deciliter, mIU/mL = milli-international units per milliliter, mlU/L = milli-international units per liter, ng/mL = nanograms per deciliter, pg/ml = picograms per milliliter, U/L = units per liter, units per milliliter.

Two samples of stool tested negative for occult blood. Thick and thin films for malaria were negative for the presence of any forms of parasite. The work up for amenorrhea showed normal follicle-stimulating hormone, luteinizing hormone, and serum estradiol.

In light of multi-etiology anemia, malabsorption was considered and work up for CD was started. The patient was found to have high levels of anti-tissue transglutaminase IgA (TTg-IgA) and anti-tissue transglutaminase IgG (TTG-IgG). Thus, the patient was diagnosed as a case of anemia resulting from malabsorption caused by CD. Endoscopy was not performed as per patient preference.

The patient was initiated on a GFD and received supplements containing folates, iron, calcium, zinc, and vitamins (i.e., A, D, E, B_6_, and B_12_). After 3 months of treatment and dietary modification hemoglobin level of the patient rose to 9.2 g/dL, and normalized at 1 year follow-up.

She also gained 2 kg weight at 3 months after treatment and had successive weight gain thereafter. Her menstrual cycle was restored 1 year after initiation of GFD.

## 3. Discussion

CD is autoimmune enteropathy in genetically susceptible individuals characterized by sensitivity to a protein called gluten. Gluten is mainly found in wheat, rye, and barley. CD has heterogeneous presentations. The presentation of CD is variable and includes hematological disorders, liver disorders, musculoskeletal disorders, dermatological disorders, neuropathy, pulmonary involvement, fatigue, abdominal pain, bloating, bowel changes, weight loss, headaches, alopecia, psychological disturbances, and male and female reproductive system problems. Undiagnosed cases of CD can lead to osteoporosis, autoimmune illnesses, and malignancy. Malnutrition can result in recurrent spontaneous abortions, unexplained infertility, and perinatal problems.^[9]^

The atypical CD is characterized by the absence of or minimal gastrointestinal symptoms, and the presence of atypical symptoms, such as iron deficiency anemia (IDA), osteopenia or osteoporosis, short stature, and infertility. In the silent form, the individuals are asymptomatic and may occasionally be diagnosed histologically or serologically. The latent forms have either the patients with previous CD diagnosis who responded to GFD with normal histology or only intraepithelial lymphocytes infiltration or individuals with normal intestinal mucosa taking a gluten diet, who will subsequently develop CD. The patients with CD not responding to GFD are listed in refractory form.^[8]^

CD is associated with infertility and has a higher rate of stillbirths and neonatal deaths. However, GFD can lead to the resolution of infertility. The prevalence of male infertility in CD is not common.^[10]^ Secondary amenorrhea was identified in patients regardless of malnutrition level in CD.^[7]^ CD is also associated with other endocrine and autoimmune diseases. Moreover, CD patients can develop liver disorders, dental enamel defects, and neurological complications. Iron deficiency anemia and megaloblastic anemia are frequent hematologic issues associated with CD.^[11]^

The cornerstone treatment for CD is a GFD. GFD therapy improves milder forms of IDA in these patients. If other causes of IDA co-exist in addition to CD, they must be treated to improve iron balance. A strict and life-long GFD is effective and safe, preventing certain potential complications, including autoimmune disease, cancer, infertility, osteoporosis, and prematurity.^[2]^ The holistic management plan should include a multidisciplinary team consisting of a comprehensive dietary plan, patient education, and continuous long-term follow-up.^[12]^

All patients with clinical suspicion of CD should undergo serologic testing. The IgA tTG has 98% sensitivity and 98% specificity for CD. In case of a negative IgA TTG antibody with suspicion of CD, a total IgA level should be done because up to 3% of patients can have IgA deficiency.^[3,13]^ Traditionally, the diagnosis of CD has to be confirmed on the demonstration of mucosal injury in the duodenal biopsy. This invasive approach has been considered necessary to ensure the diagnosis before starting GFD. The high specificity of modern serological tests and the desire to reduce the need for invasive investigations have helped gradually shift CD workup towards less invasive screening assays.^[14]^

## 4. Conclusions

The pathogenesis of the atypical CD is multifactorial. Common features of classical forms, such as bloating, diarrhea, etc., are absent. The presence of anemia, malnutrition or significant weight loss should raise a concern about CD. Proper knowledge about CD can help patients and improve their quality of life.

## Author contributions

**Conceptualization:** Arif Mumtaz, Qaisar Ali Khan, Abdul Baqi.

**Data curation:** Arif Mumtaz, Qaisar Ali Khan, Nowshad Asim.

**Formal Analysis:** Arif Mumtaz, Qaisar Ali Khan, Nowshad Asim, Sumaira Iram.

**Investigations:** Arif Mumtaz.

**Methodology:** Qaisar Ali Khan, Muhammad Junaid Tahir, Zohaib Yousaf.

**Validation:** Abdul Baqi, Abdul Majeed, Muhammad Junaid Tahir, Md. Saiful Islam, Zohaib Yousaf.

**Writing – original draft:** Qaisar Ali Khan, Nowshad Asim, Abdul Baqi, Sumaira Iram, Zohaib Yousaf.

**Writing – review & editing:** Abdul Majeed, Muhammad Junaid Tahir, Md. Saiful Islam, Zohaib Yousaf.
